# Safety of Therapeutic Apheresis in Children and Adolescents

**DOI:** 10.3389/fped.2022.850819

**Published:** 2022-04-12

**Authors:** Christina Taylan, Anne Schaaf, Corina Dorn, Claus Peter Schmitt, Sebastian Loos, Nele Kanzelmeyer, Lars Pape, Dominik Müller, Lutz T. Weber, Julia Thumfart

**Affiliations:** ^1^Pediatric Nephrology, Children’s and Adolescents’ Hospital, University Hospital of Cologne, Faculty of Medicine, University of Cologne, Cologne, Germany; ^2^Department of Pediatric Gastroenterology, Nephrology and Metabolic Diseases, Charité Universitätsmedizin Berlin, Berlin, Germany; ^3^Pediatric Nephrology, University Hospital for Pediatric and Adolescent Medicine, Heidelberg, Germany; ^4^University Medical Centre Hamburg-Eppendorf, University Children’s Hospital, Hamburg, Germany; ^5^Department of Pediatric Kidney, Liver and Metabolic Diseases, Hannover Medical School, Hanover, Germany; ^6^Department of Pediatrics II, University Hospital of Essen, Essen, Germany

**Keywords:** register, pediatric, plasma exchange (PE), immunoadsorption (IA), complications

## Abstract

**Background:**

Therapeutic apheresis (TA) is based on the principles of either removing dissolved pathogenic substances (e.g., antibodies) from the blood plasma or replacing plasma factors. It expands the therapeutic scope for a variety of diseases. Safety analysis in the pediatric field are scant. The aim of this analysis was to analyze specific complications of TA modalities – plasma exchange (PE) and immunoadsorption (IA) – in children and adolescents.

**Methods:**

Children and adolescents (*n* = 298) who had received TA from 2008 to 2018 in five pediatric nephrology centers were analyzed retrospectively. In total, 4.004 treatments (2.287 PE and 1.717 IA) were evaluated.

**Results:**

Indications for TA were mainly nephrological and neurological diseases. The three main indications were antibody-mediated graft rejection (13.4%), hemolytic uremic syndrome mainly with neurological involvement (12.8%), and AB0-incompatible transplantation (11.7%). Complications developed in 440 of the 4004 sessions (11%), of which one third were non-specific (nausea, headache). IA was better tolerated than PE. Complications were reported in 9.5% (*n* = 163) of the IA versus 12.1% (277) of the PE sessions (*p* < 0.001). When considering different types of complications, significantly more non-specific/non-allergic events (*p* = 0.02) and allergic reactions occurred in PE sessions (*p* < 0.001). More complications occurred with PE, when using fresh frozen plasma (16.2%; *n* = 145) in comparison to human albumin (14.5%; *n* = 115) (*p* < 0.001).

**Conclusions:**

Therapeutic apheresis in childhood and adolescence is a safe treatment procedure. IA showed a lower complication rate than PE. Therefore, IA may be preferably provided if the underlying disease pathomechanisms do not require PE.

## Introduction

Apheresis refers to the separation or removal of a blood component. Therapeutic apheresis (TA) is the use of apheresis to treat various disease states. Apheresis can be used to selectively remove plasma (plasma exchange PE), or specifically target antibodies (immunoadsorption IA). More and more antibody mediated diseases have been identified which may be removed by TA. Therapeutic apheresis offers an opportunity to expand the therapeutic scope in diseases across different disciplines such as nephrology, neurology, rheumatology and hematology (1–3). Indications for TA comprise rare diseases such as antibody-mediated rejections after organ transplantation, AB0 incompatible kidney transplantation, recurrence of focal segmental glomerulosclerosis, antibody mediated encephalitis or myasthenia gravis.

The basic principle of PE is the elimination of pathogenic molecules, by replacing the patient’s plasma by foreign plasma or human albumin solution (4). Another option is IA, in which specially coated columns are used to adsorb antibodies from the patient’s blood (5). While TA in adult medicine is becoming more and more evidence-based, reports in pediatrics are still scant (1, 4, 6, 7). The pediatric studies mostly consist of case reports and small size studies, since the diseases requiring TA are rare and challenges regarding the vascular access and volume of the extracorporeal circuit are substantial especially in young children (8, 9). Recently, notable improvements in device design have been achieved regarding safety and efficiency. Extracorporeal volumes decreased, which should reduce the risk of hypovolemia. Monitors and alarms have been developed to detect clots, air build-up, and hazardous line pressures. Anticoagulation has been gradually adapted to the needs of pediatric patients. The size and weight of the devices have been decreased, allowing greater mobility to treat patients all over the departments. These reasons made TA attractive for the use in pediatric centers that are familiar with extracorporeal procedures. Nevertheless, there is still a lack of data to assess the risk of TA treatment in childhood and thus to carry out a risk-benefit assessment to optimize the patients’ safety.

We now provide in a multi-center approach the first large scale analysis of the indications and safety of TA in children and adolescents, comprising both PE and IA.

## Materials and Methods

All children and adolescents aged 0–18 years who underwent IA or PE in the pediatric nephrology departments of Charité Universitätsmedizin Berlin, Heidelberg University Hospital, Hamburg-Eppendorf University Hospital, Cologne University Hospital, and Hannover Medical School between 2008/1/1 and 2018/12/31 were evaluated.

For this purpose, both digital and analog patient records were retrospectively evaluated. All patients were treated with apheresis machines using a membrane plasma separator. Predefined items were included from the start of treatment up to and including one week after the last TA session. Therefore, following data were collected: gender, indication, medication, treatment length, numbers of sessions treated, frequency, type of treatment (IA or PE) and substitute [fresh frozen plasma (FFP) or human albumin (HA)], vascular access, anticoagulation, occurrence and type of complications. One treatment cycle was defined as sessions done within one treatment period.

We categorized symptoms such as urticaria, itching and flushing as allergic reactions. Abdominal pain, nausea, and headache were rated as non-specific, non-allergic symptoms.

The statistical analysis was performed with IBM SPSS Statistics 25. After the descriptive analysis of the patient data, significance tests were performed. For this purpose, possible influencing factors (gender, age, type of treatment) on the development of complications were evaluated. Chi-Square-Test was used to test nominal scaling (gender, type of treatment, age group, complications) for significance. In a multivariable linear regression analysis, the association of complications with type of treatment, age and gender was assessed. The significance level was set at *p* < 0.05. Data are presented as median [minimum-maximum]. In the multivariate analysis, odds ratio and 95% confidence interval are given.

Informed consent for retrospective data analysis was obtained for all patients. The study has been performed in accordance to the Declaration of Helsinki.

## Results

### Study Population

Data were collected from a total of 298 patients. The demographic data of the patients are listed in Table 1. The median age at the start of the first treatment was 11 [0.0–17.9] years.

**TABLE 1:** Patients characteristics.

Variable	Value
Gender	149 female (50%)
	149 male (50%)
Median age at first treatment	11 [0–17.9] years
Median weight at first presentation	32.8 [3–93] kg
Total sessions done	4.004
Total cycles done	579
Median number of sessions per cycle	TA: 3 [1;331]
	IA: 4 [1;331]
	PE: 3 [1;245]
Vascular access per cycle in n (%)	283 central catheter (48.9%)
	80 fistular (13.8%)
	3 peripheral (0.5%)
	213 no data available (36.8%)
Treatment frequency n (%)	19 once (3.3%)
	227 daily (39.2%)
	214 every 2–3 days (37.0%)
	33 weekly (5.7%)
	7 every 2–3 weeks (1.2%)
	13 monthly or less (2.2%)
	66 no data available (11.4%)
Anticoagulation n (%)	462 unfractionated heparin (79.8%)
	20 citrate (3.5%)
	7 others (1.2%)
	90 no data available (15.5%)
Median exchanged plasma volume in ml	IA: 2.500 [1.000;8.960]
	PE: 2.500 [60;7.000]
Median length of session in min	IA: 130 [55;300]
	PE: 120 [15;350]

*TA, therapeutic apheresis; IA, immunoadsorption; PE, plasma exchange.*

Indications for TA were mainly nephrological and neurological diseases. Underlying diseases are listed in Table 2. Neurological indications increased over time (*n* = 3 in 2008; *n* = 8 in 2013 and *n* = 29 in 2017).

**TABLE 2:** Underlying disease per modality.

	TA n (%)	PE n (%)	PE with FFP in n	PE with HA in n	PE with FFP and HA in n	PE with unknown in n	IA n (%)	Both (PE and IA) n (%)
Auto-immune disease; Anti-Phospholipid Syndrome	4 (1.3)	4 (1.9)	4	0	0	0	0 (0.0)	0 (0.0)
Auto-immune disease; Lupus erythematodes	7 (2.3)	5 (2.4)	3	2	2	0	0 (0.0)	2 (7.4)
Auto-immune disease; Others*^1^	12 (4.0)	9 (4.3)	5	2	1	3	1 (1.7)	2 (7.4)
Auto-immune disease; TTP	9 (3.0)	8 (3.8)	6	1	1	0	1 (1.7)	0 (0.0)
Auto-immune disease; Vasculitis	6 (2.0)	6 (2.8)	4	0	2	0	0 (0.0)	0 (0.0)
Liver failure	9 (3.0)	9 (4.3)	7	0	0	2	0 (0.0)	0 (0.0)
Neurologic; Encephalitis	26 (8.7)	13 (6.2)	2	11	1	0	10 (16.7)	3 (11.1)
Neurologic; Guillain-Barré-Syndrome	8 (2.7)	8 (3.8)	5	2	0	1	0 (0.0)	0 (0.0)
Neurologic; Multiple sclerosis	6 (2.0)	6 (2.8)	4	2	0	0	0 (0.0)	0 (0.0)
Neurologic; Myasthenia gravis	7 (2.3)	2 (0.9)	0	1	1	1	4 (6.7)	1 (3.7)
Neurologic; Others*^2^	20 (6.7)	11 (5.2)	4	3	5	2	6 (10.0)	3 (11.1)
Renal; FSGS/Steroid resistant nephrotic syndrome^#^	16 (5.3)	7 (3.3)	2	1	6	4	3 (5.0)	6 (22.2)
Renal; HUS	38 (12.8)	37 (17.5)	11	18	4	4	1 (1.7)	0 (0.0)
Renal; IgA-Nephropathy/IgA Vaculitis (Purpura Schoenlein Hennoch) Nephritis	4 (1.3)	4 (1.9)	0	2	2	0	0 (0.0)	0 (0.0)
Renal; Others*^3^	9 (3.0)	7 (3.3)	2	3	2	0	2 (3.3)	0 (0.0)
Renal; RPGN	9 (3.0)	7 (3.3)	3	1	1	2	2 (3.3)	0 (0.0)
TX related; AB0 incompatible TX	35 (11.7)	23 (10.9)	11	6	4	7	7 (11.7)	5 (18.5)
TX related; Antibody-mediated rejection post TX^+^	40 (13.4)	21 (10.0)	6	9	4	6	15 (25.0)	4 (14.8)
TX related; Others*^4^	9 (3.0)	7 (3.3)	3	3	1	0	1 (1.7)	1 (3.7)
Others*^5^	9 (3.0)	6 (2.8)	3	1	0	2	3 (5.0)	0 (0.0)
n/a	15 (5.0)	11 (5.2)	8	2	1	0	4 (6.7)	0 (0.0)
Total	298 (100)	211 (100)					60 (100)	27 (100)

*TA, therapeutic apheresis; IA, immunoadsorption; PE, plasma exchange; TTP, thrombocytic thrombopenic purpura; FSGS, focal segmental glomerulosclerosis; RPGN, rapid progressive glomerulonephritis; TX, transplantation.*

*^1^Auto-immune disease; Others (n = 12): autoimmune myelopathy (n = 2), all other diagnosis each (n = 1).

*^2^Neurologic; Others (n = 20): neuromyelitis optica spectrum disorder (n = 5), myelitis transversa acuta (n = 3), chronic inflammatory demyelinating polyradiculoneuropathy (n = 2), all other diagnoses each (n = 1).

*^3^Renal; Others (n = 9): membranoproliferative glomerulonephritis (n = 2), all other diagnoses each (n = 1).

*^4^TX related; Others (n = 9): multiple organ failure after autologous stem cell (n = 2), all other diagnoses each (n = 1).

*^5^Others (n = 9): acute lymphatic leukemia (n = 2), all other diagnoses each (n = 1).

*^#^ n = 6 patients recurrence of FSGS after Tx.*

*^+^n = 3 patients had rejection and recurrence of FSGS.*

### Treatment

In total, 4004 TA sessions could be evaluated. Of these, 2287 PE (881 with FFP, 793 with HA, 204 with FFP and HA, 409 with unknown substituent) and 1717 IA were performed between 2008 and 2018 (see Table 3). 1459 PE sessions were performed in female and 828 PE session in male patients. Regarding IA, 1235 sessions were performed in female and 482 sessions in male patients. Most patients received a central venous catheter and were anticoagulated with unfractionated heparin (see Table 1).

**TABLE 3:** TA sessions done per year.

Year	IA in n	PE in n	Total n per year
2008	12	109	121
2009	0	159	159
2010	102	104	206
2011	316	154	470
2012	11	449	460
2013	197	107	304
2014	150	280	430
2015	461	99	560
2016	142	361	503
2017	157	353	510
2018	169	112	281
Total	1.717	2.287	4.004

*IA immunoadsorption, PE plasma exchange.*

When PE was used, 3 [1–245] sessions of treatment with a medium length of 120 min [15–350]. In case of using IA, on median, patients received 4 [1–331] applications with a median length of 130 min [55–300]. Most frequently, patients received TA daily (39%) or every 2–3 days (37.8%) (see Table 1).

### Complications

In 4004 TA performed, 466 complications occurred in 11.0% (*n* = 440) of sessions (see Table 4). Among these, adverse events occurred significantly less when IA was used (9.5%; *n* = 163) in comparison to PE (12.1%; *n* = 277) (*p* < 0.001).

**TABLE 4:** Complications according to treatment modality.

	TA n (%)	IA n (%)	PE n (%)	p
Allergic	58 (1.5)	3 (0.2)	55 (2.4)	< 0.001
Catheter dysfunction	83 (2.1)	56 (3.7)	27 (1.2)	< 0.001
Clotting/bleeding	65 (1.6)	29 (1.7)	36 (1.6)	0.78
Non-specific/non-allergic	146 (3.6)	49 (2.8)	97 (4.2)	0.02
Others	114 (2.8)	32 (1.9)	82 3.6)	0.001
Total n of complications	466	169	297	< 0.001
Total n of sessions done (%)	4.004 (100.0)	1.717 (42.9)	2.287 (57.1)	
Sessions with complications	440 (11.0)	163 (9.5)	277 (12.1)	

*TA, therapeutic apheresis; IA, immunoadsorption; PE, plasma exchange.*

Above all, when using PE, significantly more non-specific/non-allergic events (*p* = 0.02), allergic reactions (*p* < 0.001) and other complications (*p* = 0.001) occurred as compared to IA. Catheter dysfunctions (*p* < 0.001) were significantly more documented, when IA was performed. No correlation was shown between clotting/bleeding (*p* = 0.78) and the modality (see Table 4).

When considering PE, the different substitutes (FFP versus HA) had a significant (*p* < 0.001) impact on the complication rate. In 881 PE-sessions using FFP, 143 (16.2%) complications were documented. When HA (*n* = 793) was substituted, complications occurred in 115 sessions (14.5%). When using FFP significant more allergic reactions (*p* < 0.001) occurred. When HA was used, more catheter dysfunction (*p* = 0.006) were noticed (see Table 5).

**TABLE 5:** Complications according to substitute.

	PE with FFP n (%)	PE with HA n (%)	*p*
Allergic	33 (23.1)	5 (4.4)	< 0.001
Catheter dysfunction	13 (9.1)	12 (10.4)	0.006
Clotting/bleeding	10 (7.0)	24 (20.9)	0.48
Non-specific/non-allergic	44 (30.8)	42 (36.5)	0.85
Others	43 (30.1)	32 (27.8)	0.49
Total n of complications	143	115	< 0.001
Total n of sessions done (%)	888 (52.8)	793 (47.2)	

*PE, plasma exchange; FFP, fresh frozen plasma; HA, human albumin.*

**FIGURE 1:**
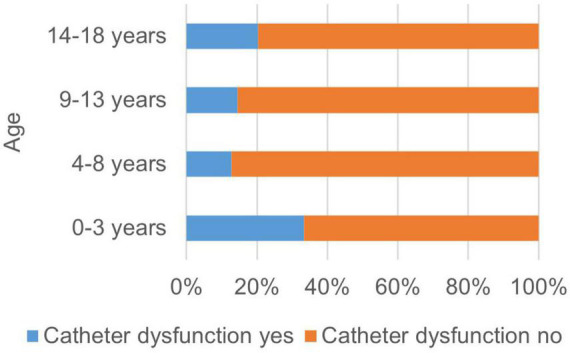
Occurrence of catheter dysfunctions related to the age of the patient. The occurrence of catheter related problems was significantly associated with younger age (*p* = 0.03).

Possible factors influencing the occurrence of complications besides modality were also investigated. In the univariate analysis, higher age was associated with a significantly higher risk for the general occurrence of complications (*p* = 0.003). The occurrence of catheter related problems was significantly associated with younger age (*p* = 0.03) (see Figure 1). There was no correlation found between age and non-specific/non-allergic events (*p* = 0.06), allergic reactions (*p* = 0.42) and clotting/bleeding (*p* = 0.09).

In the multivariate analysis, IA was associated with a significant decreased risk of the occurrence of complications (see Table 6). Age over 14 increased the risk of complications in comparison the age between 9 and 13 years. Female gender was associated with a higher general complication rate (*p* = 0.03) in the univariate analysis. No correlation between female gender and a specific type of complications was found: catheter dysfunctions (*p* = 0.08), non-specific/non-allergic events (*n* = 0.39), allergic reactions (*p* = 0.43), clotting/bleeding (*p* = 0.44) and other complications (*p* = 0.52). In the multivariate analysis, the gender effect could not be confirmed.

**TABLE 6:** Multivariate analysis of occurrence of complications.

	No complications			
		Odds ratio	95% confidence interval	p
Modality (reference PE)	IA	1.3	1.06–1.59	0.01
Age (reference 9–13 years)	0-3 years	0.83	0.55–1.25	0.37
	4-8 years	0.75	0.55–1.01	0.06
	14-18 years	0.73	0.58–0.92	0.007
Gender (reference male)	Female	0.95	0.76–1.18	0.64

*IA, immunoadsorption; PE, plasma exchange.*

## Discussion

To assess safety and efficacy of a medical procedure randomized prospective clinical trials are the gold standard. The extremely low prevalence of the diseases requiring TA in childhood and adolescents makes such studies quite unlikely and systematic analyses are usually retrospective in nature. To increase the validity of our safety analysis, we included pediatric patients treated over a period of eleven years in six large German pediatric nephrology centers and identified nearly 300 patients with 4.004 TA procedures, which to our knowledge presents by far the largest pediatric cohort studied up to now.

Main indications for TA were similar to previously published studies and included antibody-mediated allograft reaction, hemolytic uremic syndrome and AB0-incompatible transplantation (1, 6, 10). The indications for TA changed over time. TA treatment of hemolytic uremic syndrome has been decreased over the years since the availability of specific complement blockers. For Shiga toxin associated hemolytic uremic syndrome, TA is no longer indicated. Neurological autoimmune diseases have increasingly being treated with TA. This is in accordance to international guidelines in which TA is recommended in pediatric autoimmune encephalitis early in severe disease manifestation (11).

Based on our results, it can be emphasized that TA is a safe procedure with regard to complications including undesirable side effects in children and adolescents. Complications occurred in 11% of sessions. The complication rate is in accordance with previously published pediatric studies where complication rate varied from 6 to 19% (4, 6, 12–17). Approximately one third of complications were mild non-specific symptoms like nausea or headache comparable to other studies (1, 10, 12, 18). Neither in our study nor in other studies there was any further evidence of procedure-associated complications leading to death or major adverse sequelae.

These published studies are quite heterogeneous concerning the analyzed modalities. While Sik et al. (4) and Runowski et al. (12) included only PE, Paglialonga et al. (6) included double filtration plasmapheresis and IA as well. Paglialonga et al. analyzed adverse events in IA and double filtration plasmapheresis sessions together in comparison to PE sessions (6). They found a tendency to more adverse events in PE; however, the effect was not statistically significant.

In our study, complications occurred significantly less when IA was used in comparison to PE. When considering different types of complications, significantly more non-specific symptoms and allergic reactions occurred in PE sessions. This finding may be mainly explained by the fact that in IA procedures no foreign plasma proteins with allergic potential are infused. We could also observe significantly more complications when using FFP in comparison to HA in PE sessions reflecting the impact of foreign plasma proteins. As shown in Table 2 PE and IA was quite similar used for the different underlying diseases. Considering the different substitutes in PE, besides the two diagnosis liver failure and encephalitis there was no preferred substitute dependent on the underlying disease. Therefore, we assume that the adverse events are related to the TA method and to the substitute and not the disease itself. Some of the symptoms classified as non-specific by the centers may still reflect mild allergic reactions. Nausea and abdominal pain may represent clinical signs of non-specific reactions to the extracellular circuit but also gastrointestinal manifestations of an allergic reaction. The fact that we could observe more catheter problems in IA sessions in comparison to PE sessions can hardly be explained.

Considering the lower rate of side effects, IA should generally be preferred over PE. In most pediatric guidelines, PE is rather recommended because of study evidence (11). Furthermore, IA is not yet used in many countries, especially in younger children and treatment costs are usually higher in comparison to PE. When using PE, human albumin should be the preferred substitute unless plasma is needed.

Regarding catheter related problems, the youngest children showed more complications than older ones. However, the oldest children in our cohort showed the most procedure-associated overall adverse events. Mild non-specific symptoms could have been expressed more explicitly in this age group. Older children and adolescents may have been sensitized to the perception of symptoms, described while explaining the procedure to the family. On the other hand, the low complication rate in small children may be explained by the extended experience of the centers in extracorporeal treatment in this age group.

There are obvious limitations in this study. Due to the retrospective setting, not all parameters could be evaluated in every patient. Documentation forms differed from center to center and complications were only listed in the patient chart. No standardized documentation of the TA procedures was performed and complications were not predefined. However, complications during TA sessions are usually well documented because they often lead to changes in the procedure regarding technical parameter (e.g., blood flow reduction) and may require additional medication (e.g., antiallergic treatment).

The disease spectrum was very heterogeneous. Therefore, we could neither evaluate whether underlying disease has an influence on adverse events nor impact of TA modality in different diseases. We were also not able to score disease severity of the single patients due to lacking documentation. Disease severity may influence complication rate of TA. Follow up data of the patients were out of the scope of this study. Therefore, effectiveness of TA could not be analyzed.

In conclusion, our analysis of more than 4000 TA sessions demonstrates a high safety of both PE and IA across a large diseases spectrum. The side effects were mostly minor events. Due to the lower rate of side effects, IA should be preferred over PE in children and adolescents. Establishing a prospective TA registry should enhance data quality and allow for further conclusions on safety and efficacy of TA in the vulnerable pediatric patient groups.

## Data Availability Statement

The raw data supporting the conclusions of this article will be made available by the authors, without undue reservation.

## Ethics Statement

The studies involving human participants were reviewed and approved by Charité Universitätsmedizin. Written informed consent to participate in this study was provided by the participants’ legal guardian/next of kin.

## Author Contributions

CT, LW, DM, and JT: conceptualization. AS, CD, CS, SL, NK, and LP: methodology. CT and JT: validation. AS: formal analysis and visualization. CT, AS, and JT: writing-original draft preparation. CS, SL, NK, and LP: writing-review and editing. CT, LW, and JT: supervision. All authors contributed to the article and approved the submitted version.

## Conflict of Interest

The authors declare that the research was conducted in the absence of any commercial or financial relationships that could be construed as a potential conflict of interest.

## Publisher’s Note

All claims expressed in this article are solely those of the authors and do not necessarily represent those of their affiliated organizations, or those of the publisher, the editors and the reviewers. Any product that may be evaluated in this article, or claim that may be made by its manufacturer, is not guaranteed or endorsed by the publisher.
